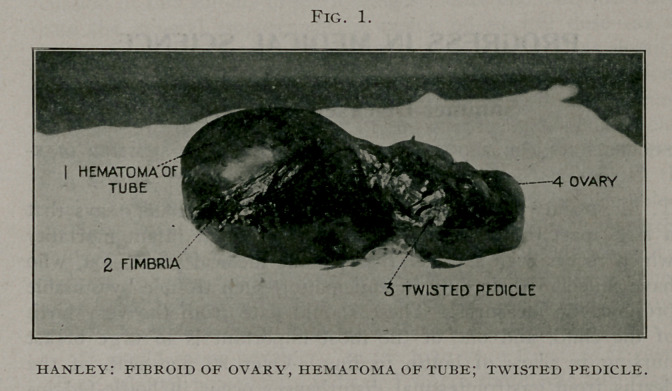# Clinical Reports

**Published:** 1905-09

**Authors:** L. G. Hanley

**Affiliations:** Buffalo, N. Y.; Surgeon to the Buffalo Hospital of the Sisters of Charity; Surgeon to the Emergency Hospital; Chief Obstetrician to St. Mary’s Infant and Maternity Hospital; Consulting Obstetrician to Erie County Hospital; Clinical Professor of Obstetrics, University of Buffalo, 428 Porter Avenue


### CLINICAL REPORTS.

### I.—Appendicitis With Erythema Exudativum Multiforme.

By L. G. HANLEY, M. D., Ph. D., Buffalo, N. Y.
Surgeon to the Buffalo Hospital of the Sisters of Charity; Surgeon to the Emergency Hospital; Chief Obstetrician to St. Mary's Infant and Maternity Hospital; Consulting Obstetrician to Erie County Hospital; Clinical Professor of Obstetrics, University of Buffalo.

John McE., age, 21; student; admitted to the hospital February 11, 1905, suffering from pain in abdomen; pain not localised but general, extending to the back. Six days prior to admission suffered greatly from constipation, when he took a cathartic, which caused him severe "cramps" with no relief from free catharsis. He has been subject to frequent attacks of constipation and accompanying the attacks a rash would appear on different parts of his body and hemorrhage from nostrils and sometimes bleeding from his mouth would occur. He says that he has always been well with the above exception. His appearance is that of one under tone. He is thin, chlorotic, tired and unable to walk without extreme exertion.

February 12, one day after his admission, he was completely prostrated, unable to leave his bed; temperature, 103; pulse, 120; leukocytosis, 21,000; nausea; urine negative; pain, which was general over the abdomen, can only be elicited over region of the appendix, and then only upon deep pressure. There is no spasm of the abdominal muscles. Patient prepared for operation. The appendix was four inches long and one quarter to one-third inch thick. There were no adhesions but the mucosa was red as scarlet. There were four enteroliths, resembling gallstones and which upon examination contained cholesterin ($C_{26}H_{44}O$). These concretions were as large as small peanuts. The cut surfaces bled very freely.

The second day after operation there was hemorrhage from the rectum, posterior nares and a rash of scarlet appearance, similar to what he has formerly experienced, covered his chest, arms and abdomen. Temperature, 104; pulse, 124; headache. Forty-eight hours after, rash had disappeared, but there was still some bleeding from the gums and rectum. He left the hospital seventeen days after operation. I saw him June 15, 1905, and he informs me that he feels well; has had no return of bleeding or rash; has gained twelve pounds in weight; is free from constipation and says he is steadily gaining.

### II.—Fibroid of Ovary, Hematoma of Tube; Twisted Pedicle.

Miss S., age 19; deaf and dumb; virgin. History of patient good; never had had any previous sickness; regular menstruation, beginning at fourteen years of age. Patient had been sick three days prior to admission to hospital.

There is a mass on left side, painful on pressure—slightly movable; motion accompanied with pain. There is a localised peritonitis; temperature, 103½; pulse, 128; leukocytosis, 17,000.

The diagnosis of tumor, with twisted pedicle, was made. Operation performed. Incision made through the median line; tumor removed, which proved to be a fibroid of the ovary 2x3 inches, with a hematoma of tube 7x3 inches. Pedicle consisted of twist of the tube near the uterus and which had nearly amputated itself. There was also about a quart of dark sanguineous fluid in the abdominal cavity; peritoneum somewhat thickened. Patient said that she had noticed that swelling for eight months, and when lying on her left side, tumor would move and cause

FIG. 1.

HANLEY: FIBROID OF OVARY, HEMATOMA OF TUBE; TWISTED PEDICLE.

her pain. Patient was operated June 1, 1905. Left hospital June 15, 1905.

Photograph shows a large fimbria, hematoma of tube, fibroid of ovary.

**III.—Fibroid of Ovary With Pregnancy; Strangulation of Bowel.**

Mrs. S., age 40; Irish; primipara; was taken suddenly sick; sharp pain in the abdomen; vomiting; December 29, 1904. Came under my care December 30, 1904. Patient about four months pregnant. Pain, which was severe at first, has greatly increased; abdomen greatly distended; vomiting; stercoraceous; pulse, 140; temperature, 99°.

On examination of abdomen a mass about the size of a child's head was present in the left iliac region. This shows very prominently regardless of the extreme distension of abdomen. Obstruction of the bowel dependent upon this tumorous mass was diagnosticated. Patient prepared for operation. An incision was made over sight of swelling and not in the median line. A fibroid of the ovary, 5x4½ inches in thickness, with a piece of omentum attached and a knuckle of bowel constricted by the omentum was discovered. The omentum was ligated, bowel liberated, which showed greatly the effects of strangulation, and was nearly gangrenous, and tumor removed. There were no bad symptoms following this operation. Patient sat up on the fourteenth day.

June 1, 1905, I delivered this woman of a dead baby. She came to the hospital claiming that she had not felt the movements of the fetus for two weeks. The child weighed 12½ pounds and from appearances looked as though it had been dead in utero for at least two weeks.

428 PORTER AVENUE.

## Figures and Tables

**Fig. 1. f1:**